# Supplementary material for: Online and Offline Behavior Change Techniques to Promote a Healthy Lifestyle: A Qualitative Study
Source: Int J Environ Res Public Health. 2022 Jan 4;19(1):521. doi: 10.3390/ijerph19010521 (PMC8744993; doi:10.3390/ijerph19010521)
Supplement: Supplementary file 1 [file ijerph-19-00521-s001.zip › File S2_Interview Guide.pdf]

File S2: Interview Guide

1) Which behavioral change techniques are used in practice?

Think of the techniques on the list you were given earlier. The list is titled: List of Behavioral Change Techniques (BCT Checklist). Could you indicate which techniques you use within the main headings. The selection of more BCTS is possible.

- You can think of setting personal goals, planning exercise activities, monitoring behavior of behavior, etc.

2) How are behavior change techniques used in online and offline coaching

- What tools do you use to encourage healthy behaviour?
- At what point in the coaching process do you use which technique?
- Do you follow the design of the combined lifestyle intervention, is that done based on your own knowledge and insight, aimed at the specific situation?
- How often are the different behavior change techniques applied per client/intervention?
- How are these techniques offered? - paper/online, monitored, self-monitoring, with/without feedback.
- In your opinion, how are the personal wishes and circumstances of a client handled within the combined lifestyle intervention?
- What determines which goals are pursued by a client?
- How do you determine behavioral change techniques to be used at a client?
- Can you tell us something about the behavioral techniques of strategies that you mainly apply 'online'?
- What determines your use of technology when guiding a client?
- For what purposes are these technologies used?
- Can you tell something about techniques of strategies that you usually apply solely 'offline'?

3) What online and offline requirements are important for the success of behavior change techniques?

- Which behavior change technique do you prefer and why?
- Which techniques do you think are the most successful and why?
- Which techniques, described in the combined lifestyle intervention, do you not use and which procedures do you do instead and why did you make this choice?
- Which techniques/strategies do you deploy per phase of behavior/or in time?
