# Supplementary material for: Online and Offline Behavior Change Techniques to Promote a Healthy Lifestyle: A Qualitative Study
Source: Int J Environ Res Public Health. 2022 Jan 4;19(1):521. doi: 10.3390/ijerph19010521 (PMC8744993; doi:10.3390/ijerph19010521)
Supplement: Supplementary file 1 [file ijerph-19-00521-s001.zip › File S3_Analysis.pdf]

File S3: Analysis

**Code System**	**1523**
Geslacht	3
Leeftijd	14
Opleiding	6
Jaren werkervaring in leefstijlcoaching	60
(neven) Werkzaamheden	4
Doelgroep	48
Soort GLI	33
Werkwijze rondom BCT's	170
Effect van gedragsveranderingstechnieken	39
Semi-gedragsveranderingstechnieken die worden gebruikt	26
BCT technieken die worden gebruikt	403
BCT's die niet of weinig worden gebruikt	65
Een grote ordner werkt niet, iets leuks, kleins wel	2
GLI online uitvoeren	152
Groepsbijeenkomsten	9
leefstijlzorg voor GLI	8
Verkeerde verwachtingen	6
Psychologische zorg tijdens de GLI	20
Wensen voor nieuwe idee binnen project	6
Uitval	2
BLUE	169
GREEN	1
MAGENTA	130
RED	104
YELLOW	43

N=14, conducted by K.B

**Code System**	
Persoonlijke gegevens	**681**
Coachingstraject	125
Gedragsveranderingstechnieken	56
Ervaringen met digitale coachingsmiddelen	206
Gebruik digitale coachingsmiddelen	156
Personaliseren	75
Overig	48
**Sets**	15
	**0**

N=14, conducted by M.B

**Code System**	**448**
Geen uitzonderingen (no special situation)	1
1) Verhouding online vs. offline	38
2) Gebruik in de praktijk	162
3) Stimuleren van gebruik e-coaching	35
4) Beinvloeden gedrag	85
5) Kenmerken interventie	87
6) Persoonlijke gegevens	40
**Sets**	**0**

N=10, conducted by D.B and M.W
